# Dietary patterns in the French adult population: a study from the second French national cross-sectional dietary survey (INCA2) (2006–2007)

**DOI:** 10.1017/S0007114516001549

**Published:** 2016-05-18

**Authors:** R. Gazan, C. Béchaux, A. Crépet, V. Sirot, P. Drouillet-Pinard, C. Dubuisson, S. Havard

**Affiliations:** Risk Assessment Department, French Agency for Food, Environmental and Occupational Health & Safety (ANSES), 94701 Maisons-Alfort, France

**Keywords:** Dietary patterns, Nutritional intakes, Food contaminant exposure, Demographic and socio-economic determinants

## Abstract

Identification and characterisation of dietary patterns are needed to define public health policies to promote better food behaviours. The aim of this study was to identify the major dietary patterns in the French adult population and to determine their main demographic, socio-economic, nutritional and environmental characteristics. Dietary patterns were defined from food consumption data collected in the second French national cross-sectional dietary survey (2006–2007). Non-negative-matrix factorisation method, followed by a cluster analysis, was implemented to derive the dietary patterns. Logistic regressions were then used to determine their main demographic and socio-economic characteristics. Finally, nutritional profiles and contaminant exposure levels of dietary patterns were compared using ANOVA. Seven dietary patterns, with specific food consumption behaviours, were identified: ‘Small eater’, ‘Health conscious’, ‘Mediterranean’, ‘Sweet and processed’, ‘Traditional’, ‘Snacker’ and ‘Basic consumer’. For instance, the Health-conscious pattern was characterised by a high consumption of low-fat and light products. Individuals belonging to this pattern were likely to be older and to have a better nutritional profile than the overall population, but were more exposed to many contaminants. Conversely, individuals of Snacker pattern were likely to be younger, consumed more highly processed foods, had a nutrient-poor profile but were exposed to a limited number of food contaminants. The study identified main dietary patterns in the French adult population with distinct food behaviours and specific demographic, socio-economic, nutritional and environmental features. Paradoxically, for better dietary patterns, potential health risks cannot be ruled out. Therefore, this study demonstrated the need to conduct a risk–benefit analysis to define efficient public health policies regarding diet.

Relationships between diet and health have already been strongly established in the literature^(^ ^1^ ^,^ ^2^ ^)^. Accordingly, the consumption of some foods (e.g. red meat, fruits, fish, alcohol, etc.) or some nutrients (e.g. SFA, Na, K, etc.) is generally associated with an increased or decreased risk of many chronic diseases such as obesity^(^ ^3^ ^–^ ^5^ ^)^, hypertension^(^ ^6^ ^–^ ^8^ ^)^, CVD^(^ ^6^ ^,^ ^9^ ^–^ ^11^ ^)^ or certain cancers^(^ ^12^ ^–^ ^14^ ^)^.

The standard approach for exploring these risk–benefit relationships has been to focus on one specific food or one nutrient without considering the diet as a whole^(^ ^15^ ^)^. However, it is necessary to consider the potential interactions or synergistic effects between foods or nutrients in order to depict the overall effect of diet on health^(^ ^15^ ^–^ ^17^ ^)^. Multidimensional approaches, such as the identification of dietary patterns, thus allow the estimation of more reliable associations between diet and health, taking into account the overall diet and its complexity^(^ ^18^ ^–^ ^20^ ^)^. Moreover, the nutritional and socio-economic characterisation of dietary patterns can be used to define practical public health policies to promote better food behaviours in specific groups of consumers^(^ ^21^ ^,^ ^22^ ^)^. From an environmental health perspective, dietary patterns can finally contribute to identify the most exposed consumers to a series of food contaminants^(^ ^23^ ^)^.

In recent years, there has been increasing interest in studying national diets using a multidimensional approach^(^ ^20^ ^,^ ^24^ ^)^. The standard approaches applied were principal factor analyses, such as principal component analysis (PCA), multi-component analysis or cluster analysis (e.g. hierarchical cluster analysis (HCA))^(^ ^18^ ^,^ ^25^ ^,^ ^26^ ^)^. In France, only a few studies have investigated dietary patterns at a national level using multifactorial methods^(^ ^27^ ^–^ ^30^ ^)^, and only one, to our knowledge, from a representative sample of the French population^(^ ^29^ ^)^. Although these studies have provided comparable results, the lack of homogeneity of the methods performed and differences in the study population make comparisons difficult. Besides, standard approaches are not really suitable for constructing dietary patterns because of the inherent structure of the data. For instance, food consumption data include a significant number of zeros because of non-consumption of certain categories of foods and only have positive values. Hence, the assumption of a Gaussian distribution may not be valid^(^ ^31^ ^)^. Moreover, the approaches usually used show poor fit because of non-negative data and the excess of zero values, generally termed ‘sparse data’^(^ ^32^ ^)^. Lee & Seung^(^ ^33^ ^)^ proposed a new latent-variable-based method, the negative matrix factorisation (NMF) method, specifically adapted to sparse and non-negative data. This method has already been proven to be effective in food risk assessment to identify dietary patterns or chemical mixtures^(^ ^23^ ^,^ ^34^ ^,^ ^35^ ^)^.

The major aim of this study was thus to identify the main dietary patterns in the French adult population using the NMF approach and the food consumption data of a nationally representative survey (the second French national cross-sectional dietary survey (INCA2)). Next, we determined their main demographic and socio-economic characteristics and assessed their nutritional and environmental profiles in order to highlight their specific features. The dietary patterns revealed in this work will thus give an overview of the different food consumption behaviours in the French adult population, according to distinct dimensions.

## Methods

### Study population

The French INCA2 survey was carried out between December 2005 and May 2007 by the French Food Safety Agency^(^ ^36^ ^)^. This cross-sectional survey was initially designed to assess food intake in a nationally representative sample of the French population. Two independent random samples of 3- to 17-year-old children and 18- to 79-year-old adults were drawn using a multistage cluster sampling technique. The complex sampling frame was established from the national census, published by the French National Institute of Statistics and Economic Studies (INSEE), and it has been described elsewhere^(^ ^37^ ^,^ ^38^ ^)^. In brief, 181 geographical units, stratified by region of residence and size of urban area, were first randomly selected with a probability proportional to size. Then, households were randomly drawn within each primary sampling unit, and two independent sampling frames were set up: one restricted to households including at least one child and the other including households with or without children. Last, within each household, either a child or an adult was randomly selected. Participation rates were 63 % for adults and 69 % for children, yielding samples of 2624 adults and 1455 children, respectively. To ensure the national representativeness of each sample, a weighting factor for unequal sampling probabilities for differential non-responses by region, agglomeration size, age, sex, occupation of the household head, size of the household and season has been assigned to each individual. These variables were selected for adjustment because of high discrepancy between their distribution among the INCA2 sample and among the French population, using an external source (Labour force survey 2005-INSEE)^(^ ^36^ ^,^ ^39^ ^)^ (distribution among the adult sample is presented in the online Supplementary Table S1). The low variability of the weighting factor for adults (mean of 1 and a sd of 0·7) demonstrated the good representativeness of the INCA2 adult sample compared with the French general adult population.

Only adults were considered in this study. As recommended by the European Food Safety Authority, under-reporting subjects (i.e. those who, voluntarily or not, under-reported amounts consumed; 26·9 % of adult sample) were identified and included in the statistical analyses^(^ ^40^ ^)^. Besides, twenty-four subjects (0·9 % of the adult sample) with an extremely low total energy intake (TEI) were excluded from the final sample (estimated from the following formula: 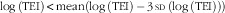 ^(^ ^41^ ^)^.

The INCA2 survey was approved by the French Data Protection Authority (*Commission Nationale de l’Informatique et des Libertés*) and the French National Council for Statistical Information (*Conseil National de l’Information Statistique*).

### Data

#### Collection of data on food consumption

Dietary intake was assessed using a 7-d food record. A trained and certified investigator delivered at home the food record with a self-administered questionnaire and explained to the subjects how to complete them. The investigator returned to the home immediately after the week to check the accuracy of the information reported in both documents. Each day of the food record was divided into three main meals (breakfast, lunch and dinner) and three between-meal snacks. The subjects were asked to describe as precisely as possible the nature and the amount of all foods and beverages consumed during the survey week. Consumed quantities were estimated using the SU.VI.MAX (*SUpplémentation en VItamines et en MInéraux AntioXydants*) photographic booklet^(^ ^42^ ^)^ or expressed directly in weight or in household measures (e.g. spoon).

Foods and beverages declared were subsequently allocated a food code including 1280 food items and were categorised into forty-three food groups and 121 subgroups. McCann *et al*.^(^ ^43^ ^)^ and preliminary analyses (data not shown) showed that the quality of the description of dietary patterns is strongly affected by the level of aggregation of foods. To obtain a satisfactory trade-off between the level of detail to discriminate individuals according to their food consumption and the difficulty in exploring a large data set by factorial analysis, the nomenclature was modified step-by-step for this study and the 1280 food items were finally reclassified into seventy-four new food groups (Table 1). This classification was based on the foods’ nutritional composition and results of previous analyses (data not shown). Eight food groups (i.e. wholegrain pasta/rice/wheat, whole milk, skimmed milk, sweetened milk, low-fat cheese, dried fruit, nectar, soft drinks with fruit) with a consumption rate <10 % were excluded to avoid excessive noise in the data, which could lead to underline too particular and isolated dietary behaviours^(^ ^44^ ^,^ ^45^ ^)^.

**Table 1:** Nomenclature (food groups and consumption rate among the 2600 individuals)

Food groups	Consumption rate (%)	Food groups	Consumption rate (%)	Food groups	Consumption rate (%)
Bread product with wheat flour	94·0	Red meat	90·2	Soft drinks with fruit	4·0
Bread product with multigrain or wholemeal flour	34·7	Poultry	70·7	Sodas and colas	32·2
Breakfast cereal	17·0	Offal	15·1	Other soft drinks	21·1
Refined pasta, rice, wheat	90·7	Other meat	17·7	Alcoholic drinks	67·7
Wholegrain pasta, rice, wheat	2·9	Processed meat	17·7	Coffee	21·3
Puff pastries	46·2	Unprocessed fish	70·8	Chicory coffee	10·3
Sweetened biscuits and cereal bars	38·0	Processed fish products	16·0	Tea, herbal tea and infusions	38·2
Crackers	28·3	Crustaceans and molluscs	31·7	Pizza	35·6
Cakes and pastries	67·1	Vegetables	98·7	Quiches	22·9
Donut pancake waffle	16·1	Potatoes and other unprocessed tubers	50·1	Savoury pastries not fried, not breaded	11·2
Whole milk	4·0	Processed potato products	77·1	Savoury pancakes, blinis, quenelles	12·2
Semi-skimmed milk	43·0	Pulses	29·5	Processed fried or breaded product	29·6
Skimmed milk	5·2	Fruits	84·5	Sandwiches	38·9
Sweetened milk	7·9	Dried fruits	9·8	Soup and stock	49·4
Regular-fat cream	24·7	Grains and nuts	20·5	Mixed dishes with meat	38·6
Yogurt and cottage cheese 0 % fat	74·3	Ice cream and sherbet	30·7	Cereal-based mixed dishes	32·9
Yogurt and cottage cheese 20 % fat	54·1	Chocolate and chocolate confectionery	49·6	Mixed dishes with vegetables	14·1
Yogurt and cottage cheese 30–40 % fat	55·9	Sugar and derivatives (not substitutes)	66·1	Mixed salad	22·3
Regular-fat cheese (not low-fat)	88·7	Non-chocolate confectionery	13·1	Dairy dessert	52·3
Low-fat cheese	8·3	Regular honey and jam (not reduced sugar)	45·2	Processed fruit	30·7
Eggs	61·2	Reduced-sugar jam and confectionery	12·9	Hot sauces	54·4
Butter and other animal fat	73·3	Bottled water	70·3	Condiment and cold dip, reduced fat	11·8
Oil	81·4	Tap water	66·4	Regular fat condiment and cold dip (not low-fat)	79·6
Margarine and other vegetable fat	19·5	Fruit juice	51·5	Herbs and spices	41·0
Low-fat spread (vegetal or animal)	45·7	Nectar	2·4		

### Individual characteristics

Individual demographic and socio-economic variables were collected using face-to-face questionnaires and self-reported data. Questionnaires provided information on individual occupational status, education level and household wealth. Household wealth was defined through questions on the household income and other related variables such as ‘having gone away on holiday for more than 4 d within the last 12 months’, ‘the number of cars in the household’, ‘the number of domestic electrical appliances’, ‘how the financial situation is perceived’, ‘financial access to desired food products’, ‘whether the idea of lacking food would be a concern’, ‘giving up health care for financial reasons’ and ‘housing occupancy status’. A wealth index was derived from a correspondence analysis as already done by Fillol *et al*.^(^ ^46^ ^)^ on variables describing household wealth (cited above). From the correspondence analysis, the score of each subject on the first principal component was used as the summary wealth index, which was divided into tertiles. In addition, for this study and according to Darmon *et al*.^(^ ^47^ ^)^, an individual was considered as living in a household experiencing food insecurity for financial reasons if she/he declared not having enough to eat (often or sometimes) because of economic reasons. Respondents were also asked to report other information such as age, sex, household composition, region and size of municipality in which the household was located. The variables and associated categories are described in the supporting information (online Supplementary Table S2).

#### Nutritional composition data

Nutritional intake was estimated by matching the French Food Composition database for the year 2008^(^ ^48^ ^,^ ^49^ ^)^ to the individual food consumption data. The individual average daily intake of macronutrients (i.e. total energy content, total carbohydrates, simple carbohydrates, total fats, SFA, proteins, alcohol, fibres and salt), minerals (i.e. Ca, Fe, Na, Mg, K) and vitamins (i.e. vitamins A, C, E, B_1_, B_6_, B_9_) was thus determined.

#### Food contamination data

Food contamination data were provided by the Second French Total Diet Study (TDS2). The TDS2 was conducted between 2006 and 2010 to evaluate the exposure of the French population to various substances that are likely to be found in foods ‘as consumed’. This study collected 20 000 food products, representing 212 types of food, for which 445 substances of interest were investigated. Food sampling was based on the data from the INCA2 survey, covering about 90 % of dietary consumption in the adult and child populations^(^ ^50^ ^)^. The 212 foods selected were linked to the INCA2 nomenclature. Of the 445 substances analysed, ten chemical substances, for which toxicological risk could not be excluded, were considered in this study^(^ ^50^ ^)^: trace elements (i.e. Pb, Al, Cd, inorganic As, organic Hg), acrylamide, one mycotoxin (i.e. deoxynivalenol (DON) and its acetylated derivatives), polychlorinated biphenyls (PCB)/dioxins (i.e. non-dioxin-like polychlorinated biphenyls (NDL-PCB), polychlorinated dibenzo-*p*-dioxins and dibenzofurans and dioxin-like polychlorinated biphenyl) and one additive (sulphites). The individual average daily exposure levels to the ten substances were estimated by combining individual food consumption data and contamination data from the food sample analysis, considering the same hypotheses as those described in the TDS2 report^(^ ^51^ ^,^ ^52^ ^)^.

### Statistical analyses

#### Identification of dietary patterns

The NMF method was applied to the data set composed of the 2600 individual daily intake (g/d) of the sixty-six food groups. The analysis was performed on the overall adult population because similar dietary patterns were identified separately in men and women (data not shown). To account for individual weight in pattern identification, the iterative least squares (LS)-NMF algorithm developed by Wang *et al*.^(^ ^53^ ^)^ and based on that described by Lee & Seung^(^ ^33^ ^)^ was used. The goal of this factorial analysis is to summarise the information available in food consumption data into an optimal number *k* of consumption systems (CS)^(^ ^23^ ^,^ ^34^ ^,^ ^35^ ^)^. In contrast to the PCA technique, each *CS*_*k*_ in the NMF is defined as a positive linear combination of foods, which are generally associated in the same diet. Thus, all *CS*_*k*_ describe the different associations of foods within the population. For each *CS*_*k*_, each food group had a coefficient that can be interpreted as the contribution of this food group to the construction of the system *CS*_*k*_. The weight of each *CS*_*k*_ in each individual’s total diet was also determined. The diet of an individual is thus represented by a combination of different *CS*_*k*_.

To implement the NMF method, an optimal number of CS must be chosen. In this study, it was selected according to the quality of the interpretation of the CS (relevancy and ease of interpretation) and a graphical approach as done in Béchaux *et al*.^(^ ^23^ ^)^ and Sy *et al*.^(^ ^35^ ^)^. Finally, a HCA was conducted to identify individuals with similar combinations of CS, defining a dietary pattern. The scores of each individual on the CS selected were used as input to the HCA. This classical clustering method consists of a step-by-step aggregation of individuals or groups of individuals who combined the CS in a similar way^(^ ^54^ ^)^, leading to one single class that includes the entire population. The number of clusters to retain was based on the inter-cluster inertia:total inertia ratio and the interpretability of the different clusters.

For each dietary pattern, the relative contribution (%) of each *CS*_*k*_ was calculated (i.e. among individuals within the same dietary pattern, the contribution of the *CS*_*k*_ is the ratio between the sum of weights of the *CS*_*k*_ and the sum of the weights of all the CS). The CS that best describes each pattern was identified according to the V test indicator, which compares the average weight of the *CS*_*k*_ in one dietary pattern with the average weight of the *CS*_*k*_ in the whole population^(^ ^55^ ^,^ ^56^ ^)^. The *CS*_*k*_ with significant and positive V tests were used to describe dietary patterns.

#### Characterisation of the dietary patterns

Demographic and socio-economic characteristics of each dietary pattern were investigated using binomial logistic regression. Each tested model identified the main demographic and socio-economic determinants of each dietary pattern independently of the others, by comparing with the overall population. Variables considered were age, level of education, wealth index, household size, household composition, occupational status, region, food insecurity and municipality size. These factors were selected because of their significant associations with the dietary patterns in univariate analysis (data not shown), as well as the consistent associations between dietary intake and these demographic and socio-economic determinants^(^ ^57^ ^–^ ^61^ ^)^. All analyses were performed among men and women separately in order to take into account the significant interaction observed between sex and other factors (data not shown).

The mean nutrient intake was calculated for each dietary pattern. The association between nutritional intake and dietary patterns was assessed using ANOVA, and specific nutrient intake was identified by comparing the mean of each dietary pattern with the overall mean. All models were controlled for age, sex, season, TEI, level of education, wealth index, occupational status, household size, food insecurity, household composition, municipality size and region. As previously mentioned, these covariates were selected on preliminary analyses and previous studies^(^ ^57^ ^–^ ^61^ ^)^.

Diet quality indices can evaluate the overall diet of an individual based on the following: (i) nutrient indicators, which reflect the adequacy to nutritional requirements; and (ii) foods to assess the variety of food intake^(^ ^62^ ^,^ ^63^ ^)^. Three scores were selected to illustrate the overall quality of the diet: the energy density (ED) of the diet^(^ ^64^ ^)^, the mean adequacy ratio (MAR)^(^ ^65^ ^)^ and the dietary diversity score (DDS)^(^ ^66^ ^)^. The ED was used as an indicator of bad nutritional quality. Low ED has been shown to have a good nutritional quality^(^ ^67^ ^)^, and a decrease of ED of the diet is recommended by several public health authorities to prevent obesity^(^ ^68^ ^,^ ^69^ ^)^. For this study, ED was calculated for each individual with respect to the energy content (kJ/g (kcal/g)) of all foods consumed (except beverages such as water, soft drinks, alcohol, milk, coffee, tea). The mean ED was assessed for each dietary pattern. MAR was used as an indicator of good nutritional quality. The MAR represents the nutritional adequacy of the diet. Multiple versions of this index have been related to health indicators^(^ ^70^ ^)^, as well to other diet quality indexes^(^ ^71^ ^–^ ^73^ ^)^. It was calculated as the mean percentage of the French daily recommended intake for twenty keys nutrients (namely proteins, fibres, vitamins A, C, E, D, B_1_, B_2_, B_3_, B_6_, B_9_, B_12_, Ca, K, Fe, Mg, Zn, Cu, I and Se). Each ratio was truncated at 100, so that a high intake of one nutrient could not compensate for the low intake of another: 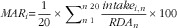 where 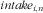 is the individual nutrient intake of the nutrient *n* and 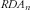 is the French RDA for the nutrient, taking into account the age and the sex of the individual^(^ ^65^ ^)^. Besides, the diet diversity is also a key element of the high quality of diets. A diverse diet increased the probability of nutrient adequacy^(^ ^74^ ^)^, and it has been associated with positive health outcomes^(^ ^75^ ^,^ ^76^ ^)^. DDS is defined as the number of specific food groups consumed over a specific period^(^ ^66^ ^,^ ^77^ ^)^. In this study, 3 d were randomly chosen for each subject: 2 weekdays and 1 weekend day. Five food groups were considered: dairy products (milk, yogurt, cheese), meat (red meat, poultry, fish and crustaceans), cereals (rice, pasta, wheat), fruits (fresh fruit, processed fruit and dry fruit) and vegetables (fresh vegetables and prepared vegetables). A food group was considered to have been consumed if at least 30 g was ingested during the 3 d. A DDS score was calculated for each individual, and it varied from 0 to 5. The mean DDS score was calculated for each pattern. Associations between dietary patterns and diet quality scores were also assessed using ANOVA adjusted for covariates, as described above. The mean of quality scores of each dietary pattern was thus compared with the overall mean.

Finally, mean contaminant exposure levels were calculated for each dietary pattern. Associations between dietary patterns and exposure levels were assessed using ANOVA-adjusted covariates described above. On the basis of the ANOVA model, specific exposure levels were identified by comparing the mean contaminant levels of each dietary pattern with the overall mean.

All values were survey-weighted means. A *P* value of 0·05 was used as the threshold of significance. All analyses were implemented in the software R version 3.0.2. The LS-NMF algorithm was implemented using the R package ‘NMF’^(^ ^78^ ^)^. The package ‘Factominer’ was used to run the clustering^(^ ^55^ ^)^. The package ‘Survey’ was used to account for the complex INCA2 sampling frame design^(^ ^79^ ^)^.

## Results

### Identification of dietary patterns

By combining graphical and interpretability criteria, seven distinct CS summarised the consumption behaviours of the 2600 individuals with respect to the sixty-six food groups. The inclusion of additional CS did not provide any further useful information for the interpretation of the dietary patterns. Moreover, additional CS were difficult to interpret, as they were composed of very few food groups (data not shown). Food groups with a score ≥2·5 % were considered as main contributors to a CS. Table 2 shows the relative contribution of the main food groups associated with each of the seven CS, designated as ‘Tradition’, ‘Snacking’, ‘Mediterranean’, ‘Simplicity’, ‘Dietetic’, ‘High-fat/sugar/salt’ and ‘Pleasant-and-convenient’ food behaviours. No strong Pearson’s correlations (<0·2715) were found between the different CS, suggesting that food behaviours related to each CS were independent of each other.

**Table 2:** Food consumption characteristics of each dietary pattern

Dietary patterns	Major CS*	%*	Major food†	%†	Major food†	%†
Small eater (23·0 %)‡	All CS contributed less than in the overall population	−	−	−	−	−
Health conscious (12·6 %)‡	Dietetic	42·7	Bread and bread products with multigrain or wholemeal flour	7·1	Vegetables	4·1
			Low-fat spread (vegetable or animal)	7·1	Low-fat condiment and dip	3·9
			Bottled water	6·6	Reduced-sugar jam and confectionery	3·8
			Soup and stock	6·3	Cakes and pastries	3·8
			Processed fruit	6·0	Tea and herbal tea	3·4
			Fruit	5·7	Crustaceans and molluscs	3·1
			Yogurt and cottage cheese (0 % fat)	5·0	Regular honey and jam	2·9
			Unprocessed fish	4·7	Coffee	2·5
Mediterranean	Mediterranean	37·2	Vegetables	12·7	Eggs	3·9
(13·0 %)‡			Oil	11·2	Yogurt and cottage cheese (30–40****% fat)	3·6
			Regular fat (not low-fat) condiment and dips	9·8	Tea and herbal teas	3·6
			Herbs and spices	7·0	Regular honey and jam (not reduced sugar)	2·5
			Tap water	6·6		
			Unprocessed fish	6·4		
			Fruit	6·3		
			Poultry	4·7		
Sweet and	Pleasant and convenient	39·7	Semi-skimmed milk	9·1	Quiches	3·5
processed			Fruit juice	6·5	Processed potato products	3·5
(13·5 %)‡			Breakfast cereal	6·3	Bottled water	3·4
			Chocolate and chocolate confectionery	5·2	Cereal-based mixed dishes	3·2
			Puff pastries	4·9	Sweet biscuits and cereal bars	2·9
			Yogurt and cottage cheese (30–40****% fat)	4·9	Processed meat	2·7
			Dairy desserts	4·6	Savoury pastries (not fried, not breaded)	2·5
			Ice cream and sherbet	4·5		
			Hot sauce	4·3		
Traditional	Traditional	44·5	Processed meat	9·5	Offal	3·1
(16·5 %)‡			Alcoholic drinks	9	Vegetables	3·0
			Regular-fat cheese	7·9	Mixed dishes with meat	2·9
			Red meat	7·0	Poultry	2·7
			Wheat bread or bread product	6·6		
			Coffee	6·0		
			Processed potato products	4·6		
	High fat/sugar/salt	7·1	Sugar and derivatives (not substitutes)	3·6		
			Crackers	16·1	Sugar and derivatives (not substitutes)	3·1
			Confectionery without chocolate, not diet	9·8	Refined pasta and rice	3·0
			Grains and nuts	9·5	Regular-fat cheese	2·9
			Other soft drinks	8·4	Dairy dessert	2·8
			Ice cream and sherbet	4·1	Sweet biscuits and cereal bars	2·6
			Savoury pancakes, blinis and quenelles	4·0	Coffee	2·5
			Processed products, fried or breaded	3·9		
			Processed fish products	3·3		
			Cakes and pastries	3·1		
Snacker (11·5 %)‡	Snacking	38·4	Sandwiches	12·6	Puff pastries	4·1
			Pizza	12·3	Processed product, fried or breaded	3·8
			Sodas and colas	11·3	Chocolate and non-chocolate confectionery	3·6
			Refined pasta and rice	6·8	Mixed dishes with meat	2·8
			Processed potato products	5·9		
			Cereal-based mixed dishes	5·3		
			Poultry	5·2		
			Red meat	4·6		
	High fat/sugar/salt	14·2	See Traditional dietary pattern			
Basic consumer (10·0 %)‡	Simplicity	40·8	Regular-fat butter and other animal fats	10·0	Honey and regular jam (not reduced sugar)	3·5
			Refined pasta and rice	8·3	Soup and stock	3·4
			Unprocessed potatoes	7·0	Regular-fat fresh cream	3·3
			Yogurt and cottage cheese (20 %)	7·0	Sugar and derivatives (not substitutes)	3·2
			Wheat bread and bread products	6·3	Processed product, fried or breaded	2·8
			Red meat	5·6	Donuts, pancakes and waffles	2·5
			Eggs	5·5	Fruit	2·5
			Pulses	4·0		
Total population	Tradition	18·6	See Traditional dietary pattern			
	Snacking	11·0	See Snacker dietary pattern			
	Mediterranean	15·2	See Mediterranean dietary pattern			
	Simplicity	17·0	See Basic-consumer dietary pattern			
	Dietetic	16·2	See Health and conscious dietary pattern			
	High fat, sugar and salt	6·9	See Traditional dietary pattern			
	Pleasant and convenient	15·0	See Sweet and processed dietary pattern			

CS, consumption system.

* CS contributing significantly more than the overall population (name and % of contribution).

† Foods contributing >2·5 % to the construction of the CS (name and % of contribution).

‡ Individuals in the population (%).

Then, seven dietary patterns with homogeneous CS combinations were identified and named according to their food consumption patterns. The major CS that best described each dietary pattern were identified and presented in Table 2. In brief, the first dietary pattern called ‘Small eater’ represented 23·0 % of the population. It consisted of consumers who used all the CS but to a lesser extent than the overall population, which means that they consumed all foods but in a lower quantity than the overall population. The second dietary pattern called ‘Health conscious’ grouped 12·6 % of the population and was characterised by individuals who used the dietetic CS significantly more than the overall population, which was mainly associated with low-fat or light foods, soups, fruits, tea and herbal tea and, paradoxically, cakes and pastries. The third dietary pattern, named ‘Mediterranean’, grouped 13·0 % of the population and was represented by individuals who used the Mediterranean CS significantly more than the overall population, which was characterised by unprocessed foods (vegetables, oil, herbs and spices, unprocessed fish, unprocessed fruit, etc.) and dairy products (condiments and cold dips (not low-fat), yogurt and cottage cheese (30–40 % fat)). Individuals in the fourth dietary pattern called ‘Sweet and processed’ grouped 13·5 % of the population. This pattern was characterised by food behaviour represented by the Pleasant-and-convenient CS characterised by an association of sweetened products such as breakfast cereals, fruit juices, chocolate bars/confectionery, dairy desserts and meals easy to prepare such as puff pastries, quiches, warm sauces, cereal-based mixed dishes, etc. The fifth dietary pattern identified as ‘Traditional’ accounted for 16·5 % of the population and was represented by individuals who followed the Tradition CS significantly more than the overall population and the High-fat/sugar/salt CS. Individuals in this pattern were therefore characterised by a consumption of foods such as alcohol (in particular wine), processed meat, cheese, bread products with wheat flour, coffee, red meat, but also crackers, confectionery without chocolate, grains and nuts, cakes and pastries, and sweetened biscuits, which characterised the High-fat/sugar/salt CS. The sixth pattern, identified as ‘Snacker’, was represented by 11·5 % of the population and was characterised by individuals who followed the Snacking CS, mainly represented by take-away products such as sandwiches, pizza, sodas and colas, puff pastries (such as ham puff pastry, ‘bouchée à la reine’, etc.) and processed foods such as processed potato products and cereal-based mixed dishes (as spaghetti carbonara, pasta gratin, etc.). This pattern also followed the High-fat/sugar/salt CS more than the overall population. The last dietary pattern called ‘Basic consumer’ accounted for 10·0 % of the population and was characterised by individuals who followed the Simplicity CS, which associated mostly simple foods such as butter/other animal fat, refined pasta/rice/wheat, unprocessed potatoes, yogurt and cottage cheese (20 % fat), bread and bread products (including bread, loaf and rusk).

### Characterisation of dietary patterns

OR and 95 % from logistic regressions are detailed in the Table 3 for men and women separately. Regardless of sex, the probability of belonging to the Health-conscious and Mediterranean dietary patterns (only for men in Traditional pattern) increased with age, conversely to the probability of belonging to the Sweet-and-processed and Snacker dietary patterns. In addition, both women and men in the Health-conscious pattern were more likely to have a higher wealth index, as well as women from the Mediterranean pattern. In contrast, men in the Snacker pattern were more likely to have a relatively low wealth index. Women from the Traditional and Small-eater patterns were more likely to have a low educational level conversely to women from the Mediterranean pattern. Women belonging to the Traditional, Snacker or Health-conscious dietary patterns were more likely to live in households experiencing food insecurity, compared with women from the Small-eater, Mediterranean and Sweet-and-processed dietary patterns. Among men, individuals from the Sweet-and-processed dietary pattern were more likely to live in households experiencing food insecurity, conversely to men belonging to the Traditional and Small-eater dietary patterns. The Mediterranean and Snacker dietary patterns had a higher probability of living in large towns or cities.

**Table 3:** Demographic and socio-economic determinants of each dietary pattern by sex (Odds ratios and 95 % confidence intervals)

	Small eater			Health conscious			Mediterranean			Sweet and processed			Traditional			Snacker			Basic-consumer		
	OR	95 % CI	*P*	OR	95 % CI	*P*	OR	95 % CI	*P*	OR	95 % CI	*P*	OR	95 % CI	*P*	OR	95 % CI	*P*	OR	95 % CI	*P*
Among women																					
Age (years)			0·211			<0·001			0·002			<0·001			0·328			<0·001			0·105
18–24	1			1			1			1			1			1			1		
25–34	1·21	0·66, 2·22	0·540	2·00	0·46, 8·70	0·353	2·47	0·49, 12·35	0·272	1·11	0·58, 2·14	0·751	1·31	0·37, 4·60	0·677	0·60	0·27, 1·31	0·198	0·48	0·14, 1·67	0·251
35–49	1·32	0·74, 2·37	0·346	4·33	1·02, 18·40	0·048	5·01	1·07, 23·40	0·041	0·60	0·32, 1·15	0·125	1·75	0·52, 5·82	0·365	0·15	0·06, 0·39	0·000	0·74	0·29, 1·90	0·537
50–64	0·81	0·46, 1·45	0·488	9·54	2·50, 36·43	0·001	8·37	1·95, 35·84	0·004	0·30	0·14, 0·65	0·002	2·44	0·72, 8·30	0·152	0·03	0·01, 0·12	0·000	0·74	0·30, 1·85	0·526
65 and +	1·04	0·53, 2·04	0·913	6·49	1·72, 24·45	0·006	8·87	2·19, 35·95	0·002	0·15	0·05, 0·44	0·001	1·51	0·28, 8·27	0·635	0·00	0·00, 0·00	0·000	1·47	0·56, 3·87	0·439
Level of education			0·003			0·450			<0·001			0·660			<0·001			0·456			0·106
Primary	1			1			1			1			1			1			1		
Secondary	0·63	0·43, 0·91	0·015	1·18	0·77,1·82	0·451	2·77	1·48, 5·16	0·001	1·14	0·59,2·18	0·700	0·65	0·34, 1·22	0·178	1·04	0·44, 2·44	0·44, 2·44	0·87	0·48, 1·58	0·644
Post-secondary	0·60	0·37, 0·97	0·039	1·24	0·71,2·18	0·455	5·63	2·80, 11·32	0·000	1·24	0·62, 2·51	0·542	0·22	0·09, 0·53	0·001	0·92	0·32, 2·67	0·877	0·39	0·18, 0·87	0·021
Wealth index			0·368			0·028			0·021			0·196			0·065			0·429			0·494
1	1			1			1			1			1			1			1		
2	0·77	0·53, 1·11	0·161	1·16	0·72, 1·87	0·544	1·64	0·88, 3·08	0·120	0·75	0·47, 1·19	0·222	0·61	0·27, 1·41	0·251	1·46	0·65, 3·25	0·356	1·11	0·61, 2·02	0·721
3	0·71	0·47, 1·09	0·119	1·70	1·04, 2·80	0·036	1·73	0·89, 3·36	0·109	0·47	0·27, 0·81	0·007	0·93	0·40, 2·19	0·873	0·76	0·29, 2·02	0·582	1·02	0·47, 2·20	0·963
Household size			0·060			0·052			0·551			0·208			0·891			0·149			0·136
1 or 2	1			1			1			1			1			1			1		
3 or 4	0·68	0·42, 1·11	0·122	0·84	0·46, 1·53	0·568	1·32	0·69, 2·55	0·403	1·03	0·57, 1·86	0·923	1·17	0·41, 3·32	0·775	2·99	1·42, 6·29	0·004	0·73	0·30, 1·74	0·474
5 or more	1·08	0·56, 2·09	0·822	0·38	0·16, 0·88	0·024	1·09	0·43, 2·75	0·851	0·63	0·26, 1·54	0·308	1·26	0·34, 4·66	0·725	3·03	0·95, 9·67	0·061	1·11	0·35, 3·46	0·862
Household composition			0·489			0·666			0·576			0·853			0·123			0·036			0·194
Single without children	1			1			1			1			1			1			1		
Single-parent family	1·41	0·78, 2·53	0·257	1·11	0·47, 2·65	0·807	0·59	0·23, 1·50	0·269	0·84	0·40, 1·77	0·649	2·60	1·11, 6·12	0·029	0·36	0·13, 0·98	0·047	1·69	0·62, 4·63	0·306
Couple without children	1·30	0·89, 1·88	0·171	0·97	0·64, 1·48	0·903	0·77	0·48, 1·25	0·289	1·07	0·64, 1·79	0·789	2·09	0·90, 4·84	0·086	0·54	0·24, 1·21	0·135	0·75	0·40, 1·41	0·373
Couple with children	1·44	0·80, 2·62	0·228	1·24	0·58, 2·67	0·576	0·69	0·28, 1·73	0·432	0·99	0·49, 1·98	0·970	1·27	0·52, 3·15	0·598	0·31	0·12, 0·76	0·011	1·94	0·55, 6·82	0·300
Occupational status			0·324			0·169			0·490			0·868			0·741			0·578			0·005
Low	1			1			1			1			1			1			1		
Medium	0·73	0·46, 1·14	0·169	1·08	0·64, 1·82	0·787	0·75	0·45, 1·25	0·272	1·26	0·73, 2·18	0·410	1·28	0·62, 2·66	0·508	0·94	0·42, 2·12	0·886	2·61	1·31, 5·21	0·006
High	1·10	0·59, 2·02	0·768	1·82	0·90, 3·68	0·098	0·61	0·30, 1·26	0·181	0·87	0·40, 1·89	0·724	1·29	0·37, 4·45	0·690	1·01	0·37, 2·77	0·984	0·39	0·10, 1·57	0·187
Inactive	0·76	0·53, 1·11	0·153	1·58	0·96, 2·60	0·075	0·99	0·58, 1·70	0·975	1·01	0·62, 1·65	0·967	0·77	0·34, 1·74	0·526	0·61	0·31, 1·22	0·166	1·53	0·87, 2·68	0·143
Region			0·059			<0·001			0·011			0·139			0·301			0·409			0·077
Paris	1			1			1			1			1			1			1		
North-east	0·77	0·48, 1·24	0·283	2·06	1·07 , 3·94	0·030	0·65	0·33, 1·27	0·209	1·07	0·55, 2·06	0·850	2·16	0·70, 6·65	0·179	1·79	0·72, 4·42	0·210	0·52	0·23, 1·18	0·120
North-west	0·52	0·32, 0·84	0·007	3·24	1·68, 6·25	0·000	0·74	0·37, 1·50	0·407	1·17	0·60, 2·29	0·651	0·97	0·26, 3·63	0·959	0·94	0·39, 2·26	0·887	1·29	0·63, 2·66	0·484
South-east	0·85	0·54, 1·33	0·473	1·43	0·74, 2·73	0·284	1·53	0·85, 2·77	0·160	0·67	0·36, 1·26	0·215	1·39	0·45, 4·28	0·567	1·16	0·53, 2·52	0·714	0·65	0·31, 1·35	0·246
South-west	0·64	0·38, 1·09	0·100	3·01	1·49, 6·06	0·002	1·24	0·61, 2·51	0·551	0·55	0·27, 1·12	0·097	1·51	0·41, 5·61	0·536	0·79	0·26, 2·37	0·669	0·87	0·33, 2·28	0·772
Food insecurity			0·009			0·026			0·016			<0·001			<0·001			0·004			0·081
No	1			1			1			1			1			1			1		
Yes	0·88	0·55, 1·39	0·581	1·05	0·60, 1·86	0·857	0·72	0·34, 1·50	0·377	0·28	0·14, 0·56	0·000	1·86	0·86, 4·03	0·115	3·07	1·30, 7·26	0·011	1·71	0·87, 3·35	0·121
Municipality size			0·435			0·873			0·677			0·340			0·866			0·177			0·393
Rural	1			1			1			1			1			1			1		
Small and medium	0·78	0·49, 1·25	0·303	0·92	0·63, 1·33	0·656	1·22	0·68, 2·17	0·503	0·72	0·40, 1·29	0·272	0·80	0·33, 1·92	0·615	1·74	0·65, 4·68	0·272	1·55	0·83, 2·92	0·171
Large	0·85	0·62, 1·16	0·294	0·90	0·53, 1·53	0·690	0·93	0·58, 1·48	0·756	1·13	0·71, 1·79	0·607	0·99	0·51, 1·91	0·978	1·94	0·99, 3·79	0·054	1·13	0·68, 1·88	0·647
Among men																					
Age (years)			0·599			<0·001			<0·001			<0·001			<0·001			<0·001			0·064
18–24	1			0·00	0·00, 0·00	<0·001	1			1			1			1			1		
25–34	0·49	0·20, 1·20	0·119	1			4·48	0·43, 46·84	0·210	0·70	0·30, 1·66	0·422	8·01	2·40, 26·75	0·001	0·53	0·21, 1·35	0·180	2·65	0·70, 10·00	0·151
35–49	0·61	0·26, 1·45	0·262	2·35	0·67, 8·28	0·184	13·71	1·64, 114·57	0·016	0·37	0·17, 0·79	0·011	11·28	3·60, 35·35	<0·001	0·13	0·05, 0·33	<0·001	4·69	1·38, 15·97	0·014
50–64	0·65	0·27, 1·55	0·334	2·72	0·90, 8·22	0·077	14·65	1·99, 107·95	0·009	0·38	0·16, 0·89	0·026	16·22	5·34, 49·24	<0·001	0·01	0·00, 0·04	<0·001	4·48	1·51, 13·30	0·007
65 and +	1·25	0·42, 3·75	0·691	2·63	0·71, 9·70	0·148	15·96	2·23, 114·42	0·006	0·13	0·04, 0·44	0·001	16·18	4·99, 52·39	<0·001	0·01	0·00, 0·05	<0·001	3·83	1·17, 12·49	0·026
Level of education			0·482			0·516			0·284			0·381			0·014			0·682			0·544
Primary	1			1			1			1			1			1			1		
Secondary	0·70	0·40, 1·22	0·204	0·79	0·37, 1·67	0·540	1·38	0·63, 3·02	0·414	1·52	0·71, 3·25	0·284	1·21	0·75, 1·94	0·438	0·70	0·30, 1·63	0·407	0·96	0·49, 1·89	0·915
Post-secondary	0·64	0·33, 1·25	0·190	1·00	0·42, 2·40	0·992	1·72	0·74, 3·97	0·204	1·74	0·71, 4·26	0·229	0·78	0·45, 1·36	0·381	0·79	0·29, 2·14	0·647	1·33	0·63, 2·85	0·456
Wealth index			0·918			0·027			0·608			0·901			0·814			0·033			0·624
1	1			1			1			1			1			1			1		
2	0·91	0·53, 1·56	0·720	1·72	0·68, 4·35	0·252	1·90	0·88, 4·12	0·103	1·42	0·73, 2·77	0·303	0·81	0·51, 1·28	0·367	0·52	0·27, 0·97	0·040	1·27	0·61, 2·67	0·526
3	0·98	0·54, 1·79	0·947	2·20	0·86, 5·65	0·102	1·94	0·83, 4·55	0·129	1·34	0·63, 2·84	0·447	0·72	0·44, 1·18	0·189	0·68	0·32, 1·45	0·321	0·95	0·44, 2·06	0·898
Household size			0·349			0·355			0·273			0·445			0·391			0·274			0·306
1 or 2	1			1			1			1			1			1			1		
3 or 4	0·68	0·32, 1·47	0·326	1·43	0·62, 3·31	0·399	1·72	0·79, 3·72	0·169	0·84	0·43, 1·62	0·602	0·98	0·56, 1·70	0·938	1·04	0·50, 2·14	0·920	1·05	0·51, 2·13	0·898
5 or more	0·40	0·14, 1·18	0·098	0·78	0·19, 3·23	0·734	0·77	0·21, 2·86	0·696	1·07	0·41, 2·80	0·888	1·31	0·58, 2·93	0·513	0·80	0·28, 2·32	0·680	2·03	0·65, 6·38	0·225
Household composition			0·065			0·238			0·248			0·200			0·142			0·327			0·914
Single without children	1			1			1			1			1			1			1		
Single-parent family	0·52	0·15, 1·83	0·306	0·63	0·21, 1·96	0·428	1·33	0·37, 4·79	0·662	1·05	0·36, 3·06	0·932	3·04	1·19, 7·76	0·020	1·13	0·44, 2·94	0·797	0·62	0·16, 2·44	0·493
Couple without children	0·69	0·42, 1·13	0·137	1·34	0·69, 2·58	0·386	0·93	0·48, 1·82	0·842	0·61	0·32, 1·16	0·133	1·50	0·95, 2·36	0·079·	1·11	0·53, 2·32	0·792	0·78	0·42, 1·41	0·407
Couple with children	1·46	0·64, 3·33	0·370	0·31	0·03, 3·04	0·317	0·43	0·14, 1·32	0·140	1·47	0·58, 3·74	0·422	1·43	0·70, 2·92	0·323	0·59	0·23, 1·47	0·253	0·83	0·30, 2·32	0·721
Occupational status			0·303			0·685			0·750			0·709			0·709			0·692			0·861
Low	1			1			1			1			1			1			1		
Medium	1·09	0·64, 1·87	0·743	1·12	0·46, 2·74	0·809	0·67	0·29, 1·56	0·351	1·31	0·68, 2·51	0·419	0·85	0·53, 1·35	0·480	1·23	0·60, 2·51	0·573	1·06	0·56, 2·00	0·853
High	1·13	0·52, 2·47	0·152	1·44	0·50, 4·14	0·501	0·70	0·29, 1·65	0·890	1·35	0·60, 3·05	0·424	1·34	0·76, 2·38	0·533	0·38	0·15, 0·97	0·936	0·81	0·33, 1·95	0·502
Inactive	0·60	0·30, 1·21	0·750	1·70	0·72, 4·04	0·229	1·07	0·40, 2·85	0·413	1·33	0·66, 2·66	0·472	0·84	0·49, 1·45	0·312	0·96	0·40, 2·34	0·043	1·29	0·62, 2·69	0·632
Region			0·632			0·781			0·142			0·156			0·104			0·010			0·006
Paris	1			1			1			1			1			1			1		
North-east	0·86	0·47, 1·58	0·621	0·79	0·31, 2·04	0·630	0·74	0·29, 1·89	0·532	1·84	0·79, 4·30	0·157	1·56	0·89, 2·75	0·125	0·83	0·38, 1·80	0·634	0·47	0·23, 0·96	0·039
North-west	0·55	0·28, 1·08	0·084	1·07	0·40, 2·88	0·895	0·96	0·41, 2·21	0·917	1·65	0·73, 3·74	0·233	1·46	0·81, 2·66	0·212	0·56	0·25, 1·29	0·175	1·00	0·48, 2·08	1·000
South-east	0·71	0·40, 1·28	0·258	0·73	0·29, 1·82	0·494	1·78	0·83, 3·79	0·137	1·01	0·43, 2·38	0·991	1·79	1·04, 3·11	0·037	1·16	0·56, 2·41	0·680	0·34	0·15, 0·76	0·008
South-west	0·79	0·40, 1·59	0·513	1·05	0·39, 2·85	0·926	1·27	0·50, 3·24	0·615	1·51	0·59, 3·82	0·389	1·39	0·74, 2·59	0·302	0·38	0·14, 1·06	0·065	0·71	0·33, 1·55	0·392
Food insecurity			0·005			0·182			0·325			0·031			0·023			0·264			0·212
No	1						1			1			1			1			1		
Yes	0·67	0·32, 1·39	0·281	0·61	0·18, 2·04	0·426	1·38	0·51, 3·74	0·528	2·03	0·95, 4·36	0·069	0·68	0·35, 1·31	0·250	0·93	0·43, 2·01	0·854	1·62	0·67, 3·93	0·283
Municipality size			0·422			0·614			0·099			0·453			0·207			<0·001			0·989
Rural	1			1			1			1			1			1			1		
Small and medium	1·05	0·56, 1·96	0·888	0·72	0·36, 1·44	0·352	1·37	0·55, 3·41	0·496	1·11	0·56, 2·21	0·759	0·90	0·55, 1·49	0·690	0·85	0·33, 2·20	0·741	1·04	0·49, 2·20	0·925
Large	0·75	0·47, 1·19	0·226	0·88	0·37, 2·09	0·771	1·95	1·05, 3·62	0·034	0·75	0·45, 1·27	0·285	0·71	0·49, 1·04	0·082	3·05	1·69, 5·49	<0·001	1·04	0·62, 1·73	0·883

Nutritional intake for each dietary pattern is shown in [Table tab4]Table 4. The energy intake was lower than the overall population for the Small-eater but higher for the Sweet-and-processed, Traditional and Basic-consumer dietary patterns. These three latter dietary patterns were also characterised by higher intake of SFA, mainly because of a higher consumption of savoury or sweet pastries, chocolate for Sweet-and-processed pattern and higher consumption of animal products (i.e. butter, cream, cheese or red meat) for Traditional and Basic-consumer patterns. The Health-conscious and Mediterranean dietary patterns had higher intake of fibres than the overall population, primarily because of a higher consumption of fruits, vegetables and wholemeal bread (for Health-conscious pattern only), leading also to higher intake of many minerals and vitamins than the overall population. In addition, Sweet-and-processed pattern showed higher intake of some minerals and vitamins, probably because of a higher consumption of fruits juice and breakfast cereals (which are, for most of them, fortified). Conversely, the Small-eater, Snacker, Traditional and Basic-consumer dietary patterns showed intake of almost all mineral and vitamins studied, which was lower than the overall population. Only the Traditional and Health-conscious dietary patterns had higher intake of Na than the overall population, primarily because of a high consumption of cheese and processed meat and a high consumption of wholemeal bread and bottled water for each pattern, respectively.

Scores of nutritional quality (DDS, MAR, ED) were significantly different across dietary patterns (Table 4). Mostly because of an insufficient intake of fruits and vegetables, the Traditional and Snacker dietary patterns showed significantly lower DDS values than the overall population; 20·4 and 30·7 % of individuals from the Traditional and Snacker dietary patterns, respectively, had a DDS value of 4, and 13·5 and 6·3 %, respectively, had a DDS value of 3 (data not shown). Conversely, the Health-conscious and Mediterranean dietary patterns consumed at least 30 g of dairy products, meat, cereals, fruits and vegetables over 3 d, leading to higher DDS values than the overal population; 95 and 92 % of consumers, respectively, had a DDS value of 5 (data not shown). The MAR, a composite indicator for nutrient adequacy, was higher than the mean in the overall population for individuals from the Health-conscious and Mediterranean dietary patterns, as well as for Sweet-and-processed and Basic-consumer dietary patterns. Individuals from the Health-conscious and Mediterranean patterns, who consumed higher amounts of foods with high nutritional density and low ED, such as fruits, vegetables and unprocessed fish, had also a lower ED than the overall population. ED was higher than the mean in the overall population for the Small-eater, Traditional and Snacker dietary patterns, patterns for which the MAR was significantly lower than the overall population.

**Table 4 tab4:** Nutrient intake and diet quality indicators of each by dietary pattern (Survey-weighted mean values and standard deviations)

	Small eater		Health conscious		Mediterranean		Sweet and processed		Traditional		Snacker		Basic consumer		Total pop.		
Nutrients	Mean	sd	Mean	sd	Mean	sd	Mean	sd	Mean	sd	Mean	sd	Mean	sd	Mean	sd	*P* *
Energy (kJ/d)	6378·5	1771·9	8278·4	2199·5	8199·8	2083·6	8773·8	2244·3	10 128·6	2431·3	8527·8	2599·1	9421·9	2771·9	8346·2	2576·5	
Energy (kcal/d)	1524·5†	423·5	1978·6	525·7	1959·8	498·0	2097·0‡	536·4	2420·8‡	581·1	2038·2	621·2	2251·9‡	662·5	1994·8	615·8	<0·001
Macronutrients																	
Total fat (g/d)	58·7	19·5	72·2†	21·5	80·3‡	20·7	78·7	23·5	90·3	24·3	73·6†	21·9	85·2	28·7	75·5	25·0	<0·001
SFA (g/d)	26·3	9·5	31·4†	10·8	31·2†	10·2	37·3‡	12·0	41·0‡	12·5	33·1†	10·6	40·7‡	15·9	33·7	12·7	<0·001
Carbohydrates (g/d)	156·3	50·0	213·5‡	69·3	193·8†	63·9	231·2‡	67·5	220·5†	72·7	224·1‡	79·4	243·6‡	85·5	205·6	74·4	<0·001
Simple carbo (g/d)	64·7	25·3	96·0‡	32·1	89·0	35·0	111·7‡	38·2	78·5†	35·9	99·3‡	51·6	93·9	40·8	87·3	39·5	<0·001
Alcohol (g/d)	6·2	8·9	8·6†	11·0	8·6†	11·0	6·0†	9·2	30·7	22·8	7·4	13·8	8·6	11·8	11·2	16·1	<0·001
Proteins (g/d)	64·2†	16·6	81·2‡	19·6	79·8	22·0	82·0†	21·6	100·6	24·9	80·1†	25·3	90·0	26·3	81·2	24·9	<0·001
Fibres (g/d)	12·3†	4·2	9·7‡	6·5	18·9	5·7	15·8†	5·5	16·9†	5·8	13·6†	4·8	18·5	7·2	16·1	6·2	<0·001
Salt (g/d)	5·4	1·8	7·2‡	2·3	6·8	2·3	6·7†	2·1	8·9	2·8	6·8	2·6	7·9	2·9	7·0	2·6	<0·001
Minerals																	
Ca (mg/d)	668·2†	235·5	967·2‡	297·5	874·1	332·3	1046·5‡	295·9	921·3†	351·0	753·1†	305·8	880·1†	307·3	856·3	326·9	<0·001
Fe (mg/d)	9·4	3·7	12·8	5·7	12·5	4·7	13·2‡	5·3	14·9	4·4	11·2†	3·9	12·9†	4·5	12·2	4·9	<0·001
Na (mg/d)	2142·6	709·8	2840·9‡	911·8	2664·6	886·9	2653·4†	818·9	3508·9	1101·6	2688·7	1033·6	3098·2†	1159·5	2750·6	1031·2	<0·001
Mg (mg/d)	210·6†	63·2	316·2‡	121·1	281·7‡	79·6	281·1	75·0	313·5	94·3	243·7†	81·0	288·7†	85·9	271·2	93·9	<0·001
K (mg/d)	2143·7†	548·8	3142·5‡	821·4	2986·0‡	814·1	2896·8‡	718·5	3149·5†	749·4	2356·0†	721·1	3042·8	859·4	2760·6	837·4	<0·001
Vitamins																	
Vit A (μg/d)	910·9	683·3	1486·8‡	962·7	1402·8‡	1052·0	986·5†	549·3	1447·1	1114·6	706·2†	464·3	1294·8†	795·6	1160·9	882·1	<0·001
Vit C (μg/d)	64·2†	37·3	116·0‡	52·9	107·5‡	50·8	103·9‡	54·9	77·8†	45·1	62·2†	38·5	84·3†	46·5	85·7	50·3	<0·001
Vit E (mg/d)	8·2	4·1	12·1‡	4·8	14·1‡	6·4	10·4	4·4	11·0†	5·3	8·7†	3·8	10·5†	5·0	10·5	5·2	<0·001
Vit B_1_ (mg/d)	0·9†	0·3	1·2	0·3	1·2	0·6	1·4‡	0·5	1·3†	0·4	1·1†	0·4	1·2†	0·4	1·2	0·5	<0·001
Vit B_6_ (mg/d)	1·2†	0·4	1·8‡	0·5	1·7‡	0·6	1·8‡	0·7	1·8†	0·5	1·4†	0·5	1·7	0·6	1·6	0·6	<0·001
Vit B_9_ (μg/d)	204·1†	69·2	314·4‡	87·0	309·6‡	102·9	295·2‡	102·2	285·1†	90·7	208·6†	76·1	277·8†	86·3	265·2	97·8	<0·001
Diet quality indicators																	
Dietary diversity score	4·68	0·38	4·95	0·05	4·91‡	0·08	4·80	0·20	4·67†	0·36	4·27†	0·83	4·81	0·17	4·81	0·41	<0·001
Energy density	1·1‡	0·7	0·9†	0·5	1†	0·5	1·1	0·7	1·2‡	0·7	1·2‡	0·8	1	0·6	1·1	0·6	<0·001
MAR (% of adequacy)	69·4†	12·9	85·9‡	8·6	84‡	10·0	83·3‡	9·4	83·6†	8·7	72·8†	13·5	82·8	10·1	79·3	12·6	<0·001

Vit, vitamins; carbo, carbohydrates; pop., population; MAR, mean adequacy ratio.

* ANOVA adjusted for sex, season, level of education, wealth index, occupational status, household size, food insecurity, household composition, municipality size and region, and total energy intake (except for the variable energy), significant at *P*<0·05.

† Nutritional intake significantly lower than the overall population; significant at *P*<0·05.

‡ Nutritional intake significantly higher than the overall population.

For the ten substances considered in this study, Table 5 gives the mean exposure levels for each dietary pattern. Except for acrylamide and DON and its derivatives, the Snacker dietary pattern was significantly less exposed than the overall population for all substances studied. This result can be attributed to relatively low consumption of foods that are recognised as contributors to substance exposure. On the contrary, the Health-conscious and Mediterranean dietary patterns were more exposed than the overall population to numerous substances. For instance, these patterns showed the highest exposure level to Pb, primarily because of higher consumption of water and hot drinks. Furthermore, as a result of their higher consumption of vegetables, individuals from the Health-conscious and Mediterranean dietary patterns were more exposed to Al than the overall population. The Health-conscious dietary pattern was also more exposed to PCB-NDL, primarily because of higher consumption of fish and fish products. The Basic-consumer dietary pattern was also significantly more exposed than the overall population to Cd, because of higher consumption of bread products, and to PCB-NDL, mostly because of high consumption of butter and other dairy products. Because of their high consumption of alcohol (mainly wine), individuals belonging to the Traditional dietary pattern were more exposed to sulphites than the overall population.

**Table 5:** Contaminant exposure levels of each dietary pattern (Survey-weighted means and standard deviations)

		Main contributor food	Small eater		Health conscious		Mediterranean		Sweet and processed		Traditional		Snacker		Basic consumer		Total population		
Substance	Units	(% contribution)	Mean	sd	Mean	sd	Mean	sd	Mean	sd	Mean	sd	Mean	sd	Mean	sd	Mean	sd	*P* *
Pb	μg/kg bw per d	Alcohol (14 %), bread products (13 %), water (11 %)	0·15	0·07	0·22†	0·09	0·21†	0·09	0·18‡	0·08	0·22†	0·08	0·14‡	0·07	0·19‡	0·07	0·18	0·08	<0·001
Al	μg/kg bw per d	Hot drinks (other than coffee) (13 %), vegetables other than potatoes (11 %)	30·9‡	14·5	45·7†	20·6	42·0†	18·9	38·1	14·6	35·0‡	12·7	34·2‡	14·3	39·0	15·3	37·0	16·5	<0·001
Cd	μg/kg bw per d	Bread products (22 %)	0·12	0·05	0·17†	0·07	0·15	0·05	0·14‡	0·05	0·15‡	0·05	0·13‡	0·05	0·17†	0·06	0·14	0·06	<0·001
Inorganic As	μg/kg bw per d	Water (24–27 %), coffee (14–16 %)	0·23‡	0·11	0·33†	0·18	0·28	0·13	0·27	0·14	0·28	0·14	0·22‡	0·13	0·25‡	0·11	0·26	0·14	<0·001
Organic Hg	μg/kg bw per d	−	0·01	0·03	0·02	0·05	0·03†	0·06	0·01	0·03	0·01‡	0·03	0·01‡	0·03	0·02	0·04	0·02	0·04	<0·001
Acrylamide	ng/kg bw per d	Fried potatoes (45 %), coffee (30 %)	371	266	376	306	290‡	228	389‡	295	520†	362	547†	430	368‡	300	408	324	<0·001
DON and its derivatives	ng/kg bw/d	Bread products (60 %)	278	143	345	178	304‡	157	328‡	170	393‡	195	377†	178	409	213	340	180	<0·001
PCB-NDL	pg/kg bw per d	Fish (37 %), butter (11 %), cheese (11 %), dairy products (11 %)	1501	1551	2105†	2292	2057	2215	1555‡	1234	1686	1436	1415‡	1120	2010†	1807	1728	1711	<0·001
PCDD/F and PCB-DL	pg TEQ05/kg bw per d	Fish (20 %), butter (20 %)	0·32	0·19	0·41	0·27	0·4	0·25	0·37‡	0·18	0·39	0·2	0·32‡	0·16	0·49	0·25	0·38	0·22	<0·001
Sulphites	ng/kg bw per d, μg/kg bw per d	Wine (70 %)	104	123	115‡	115	120‡	147	85‡	117	334†	266	67‡	92	122‡	133	141	180	<0·001

bw, Body weight; TEQ, toxicity equivalent quantity.

* DON, deoxynivalenol; NDL-PCB, non-dioxin-like polychlorinated biphenyls; PCDD, polychlorinated dibenzo-*p*-dioxins and dibenzofurans; DL-PCB, dioxin-like polychlorinated biphenyl ANOVA adjusted for sex, season, level of education, wealth index, occupational status, household size, food insecurity, household composition, municipality size and region and total energy intake, significant at *P*<0·05.

† Contaminant exposure level significantly higher than the overall population.

‡ Contaminant exposure level significantly lower than the overall population; significant at *P*<0·05.

## Discussion

This study identified seven main dietary patterns in the adult population in France, with very distinct food consumption behaviours. These patterns reflected specific nutritional intake and food contaminant exposure levels, as well as particular demographic and socio-economic determinants. According to their CS composition, these patterns were named Small eater, Health conscious, Mediterranean, Sweet and processed, Traditional, Snacker and Basic consumer. The results of this study were consistent with other studies, both national and international. Indeed, the patterns reported as reproducible in the review of Newby & Tucker^(^ ^80^ ^)^ (Healthy, Western, Alcohol/Drinker, and Sweets/Dessert) are similar to some patterns we observed here. Nevertheless, although some patterns were comparable across populations (in many diverse countries and continent), there was natural variation in food consumption, which can be partly attributed to the specificity of French food culture.

First of all, two dietary patterns in particular are consistently reported in industrialised countries: one is less healthful and designated as a ‘Western-style’ pattern, and the other is more healthful and called the ‘Prudent’ pattern^(^ ^18^ ^,^ ^24^ ^,^ ^81^ ^,^ ^82^ ^)^. First, the Western-style pattern generally features high consumption of bread, red and processed meat, starchy foods and high-fat products and is relatively similar to the patterns described as Traditional and Basic-consumer in this study. However, some disparities remained. On one hand, the Basic-consumer pattern was also characterised by a higher consumption of basic and unprocessed foods (egg, unprocessed potatoes, pulses) than the overall population with relatively high consumption of dairy products (cream, yogurt and butter), which may specifically reflect an older French model^(^ ^27^ ^,^ ^83^ ^)^. On the other hand, high consumption of alcoholic drinks (in particular wine), observed in our Traditional dietary pattern, is not particularly noticed for the ‘Western’ diet. Other French studies have revealed an Alcohol/meat dietary pattern, but distinctive only in its amount of alcohol and meat consumed^(^ ^28^ ^,^ ^84^ ^,^ ^85^ ^)^. Our Traditional pattern seems to reflect at least one aspect of the French culinary culture, with its strong attachment for conviviality, and pleasure of eating^(^ ^83^ ^,^ ^86^ ^)^. The dietary behaviours of these two ‘Western-like’ dietary patterns led to less healthy nutritional intake, with high energy and SFA intake and low vitamins and minerals intake. Individuals from these patterns were likely to have a lower socio-economic status. The results tend to support the assumption, often reported in the literature, that consumption is strongly influenced by socio-economic status and notably confirm a strong relationship between a higher consumption of energy-dense foods (such as fried products, cereals, potatoes, meat and meat products) and a lower socio-economic status^(^ ^87^ ^–^ ^89^ ^)^. Second, the Mediterranean and Health-conscious patterns were comparable to the ‘Prudent’ pattern, commonly identified in the literature. The name Mediterranean was chosen following the definition of a Mediterranean diet in the literature, such as a high consumption of whole grains and carbohydrates, fruits, vegetables, fish, olive oil, legumes and low to moderate amounts of saturated animal fats, red meat and wine^(^ ^90^ ^,^ ^91^ ^)^. Effectively, our Mediterranean pattern was characterised by a high consumption of fruits, vegetables, fish and oil, of which 56 % was olive oil (against 52 % in the overall population). Moreover, the Mediterranean Diet Score proposed by Trichopoulou *et al*.^(^ ^92^ ^)^ has been calculated for each individual and confirmed the existence of this Mediterranean pattern among the French adult population (data not shown). Nevertheless, our Mediterranean pattern was not characterised by a high consumption of legumes and whole-grain products, as described in the literature^(^ ^90^ ^,^ ^91^ ^)^. Similar patterns have also been identified in other French studies^(^ ^28^ ^,^ ^29^ ^,^ ^84^ ^)^, but which were also characterised by high consumption of breakfast cereals, which was not observed in this study. The Health-conscious pattern describes individuals who ate more dietetic products. Few studies have identified a group of consumers characterised by high consumption of dietary products^(^ ^93^ ^,^ ^94^ ^)^. The consumption of diet products appeared long before the INCA2 study – that is in the 1980s^(^ ^95^ ^)^; thus, the identification of such a pattern was probably because of the level of aggregation of foods chosen in this study, which identified diet products separately. Consumers in both these dietary patterns seemed to have the most nutritious dietary behaviour with a nutrient-dense diet, a higher MAR and higher consumption of foods with low ED. These dietary patterns were associated with higher socio-economic status, which support the association between a higher socio-economic status and so-called healthy foods, such as wholemeal cereal-based products, fruits and vegetables or fish already identified in the literature^(^ ^87^ ^,^ ^88^ ^)^.

In addition, we identified two patterns (Snacker and Sweet and processed) characterised by a high consumption of processed and modern foods (i.e. easy to prepare and to eat). Only one such pattern per study has generally been reported in the literature, either under the name of Processed/Unhealthy foods, characterised by the high consumption of high-energy beverages and savoury snacks^(^ ^29^ ^,^ ^93^ ^)^, or Sweets^(^ ^96^ ^–^ ^98^ ^)^, with a high consumption of dairy desserts and sweet products. These two profiles were both characterised by high energy intake and SFA intake. Conversely to the description of the pattern ‘Sweet foods and breakfast cereal’ identified by Hearty *et al*.^(^ ^93^ ^)^ among Irish adults, individuals in the Sweet-and-processed dietary pattern also had higher intake in some vitamins and minerals than the overall population, probably because of a higher consumption of fruits juices and fortified breakfast cereals. In both these dietary patterns, they were more likely to be younger, which confirms the negative association observed by Adams & White^(^ ^99^ ^)^ between age and energy from ultra-processed foods (i.e. ready-to-eat, convenient and accessible foods such as breakfast cereals, biscuits, mixed dishes, pizza, etc.).

Finally, the Small-eater dietary pattern in our study was characterised by a significantly lower consumption of all foods compared with the overall population, with lower intake of micronutrients. To our knowledge, only two studies identified a similar pattern, but these studies were performed among an elderly population^(^ ^100^ ^,^ ^101^ ^)^. In our study, although no association was observed between this dietary pattern and age, individuals belonging to the Small-eater pattern had a tendency to be older (11, 17, 32, 21 and 19 % of individuals from the Small-eater pattern were 18–24, 25–34, 34–49, 50–64 and >64 years old, respectively; data not shown). Because the presence of under-reporters might have suggested potential bias, their distribution was studied. In fact, under-reporters did not represent the majority of individuals from this pattern (48 % of individuals from the Small-eater pattern were identified as under-reporters), and, consequently, those individuals who had lower energy intake than the overall population could be considered as real small consumers.

As reported in other studies, dietary patterns can highlight the specific food habits, preferences and availability of the countries^(80,102)^. The multiplicity of dietary patterns identified in this work clearly reflects the contradictory attitudes of the French population toward food, such as health awareness, indulgence, pleasure, conviviality, but also convenience and practicality^(^ ^103^ ^,^ ^104^ ^)^. These different food consumption behaviours were also noticeable in the BMI, as defined by the World Health Organization^(^ ^5^ ^)^. For instance, profiles with ‘healthy’ food behaviour (i.e. the Health-conscious and Mediterranean patterns) had a lower proportion of individuals considered as overweight (32·8 and 29·1 %, respectively) or obese (13·0 and 10·9 %) than the more ‘unhealthy’ profiles, such as the Traditional pattern (44 % of overweight and 15·5 % of obese individuals).

One original aspect of our work was to focus, in addition to the nutritional quality of the diet, on food contaminant exposure levels of each dietary pattern. Whereas Health-conscious and Mediterranean dietary patterns seemed to have healthy dietary behaviours, these two groups of consumers seemed to be more at risk for exposure to some chemical substances. In comparison with health-based guidance values (HBGV)^(^ ^51^ ^)^, the Health-conscious pattern was considered to be at risk for its exposure to Pb, Cd, inorganic As and Al, and the Mediterranean pattern was identified to be at risk for its exposure to Pb, inorganic As, organic Hg and NDL-PCB. Conversely, the Snacker pattern had a higher ED, a lower MAR and, in comparison with recommended nutrient intake values, the highest prevalence of inadequate nutrient intake (data not shown). However, according to the HBGV, its exposure to the ten substances studied was not considered to match at-risk levels (except for acrylamide exposure). Finally, our results suggest that diets should be analysed further according to a risk:benefit ratio. Unfortunately, no comparison can be made here because, to our knowledge, to date, no other study in the literature has characterised the dietary patterns by levels of contaminant exposure.

Otherwise, this study shows that the novel factorial analysis used, the NMF, was well adapted to determining dietary patterns and successfully summarised the precise variability of food consumption in a given population. Moreover, by using an appropriate algorithm, this is the first study on this topic for which individual sampling weight was taken into account in the NMF to be representative of the French adult population^(^ ^23^ ^,^ ^34^ ^)^. In contrast to PCA, for which dietary patterns are constructed based on an opposition of ‘foods consumed’ and ‘non-consumed’, the NMF constructs food behaviour patterns using only a positive association of foods, which may better reflect reality. In addition, although it is well known that food consumption is a multidimensional phenomenon, classical factorial analysis approaches mean that one dietary pattern corresponds to one common underlying dimension (factor) of food consumption^(^ ^18^ ^,^ ^28^ ^)^. With NMF, one dietary pattern can be represented by different CS. Thus, as an example, consumers from the Traditional dietary pattern were characterised by foods composing the Traditional CS (processed meat, alcoholic drinks, coffee, etc.) but also the High-fat/sugar/salt CS (grains and nuts, crackers, etc.). Moreover, our study used different levels of aggregation of foods and distinguished some dietary patterns that were previously confounded in other studies and provided a better characterisation of those patterns. For instance, Bertin *et al*.^(^ ^29^ ^)^ identified five dietary patterns named Traditional, Diversified, Processed, Prudent and Sandwiches using a PCA based on the average frequency of consumption of forty-three food groups from the INCA2 survey. According to the foods that characterised the dietary patterns, the Processed pattern was similar to the Snacker and Sweet-and-processed patterns of this study, and the Prudent pattern was similar to the Health-conscious and Mediterranean dietary patterns. Furthermore, the Processed pattern identified by Bertin *et al*.^(^ ^29^ ^)^ differed from the overall population only by the higher consumption of sandwiches and lower consumption of other foods, whereas our Snacker dietary pattern included individuals who consumed higher quantities of several foods (sandwiches, pizza, sodas and colas, processed potato products, etc.) than the overall population. These differences further demonstrate that the NMF provides a better characterisation of the different food consumption behaviours.

Another major strength of this study was that it was based on two robust national studies. First, the INCA2 survey was conducted on a large and representative sample of the French adult population using a complex sampling frame design, with a robust collection of dietary intake using a 7-d food record, as well as numerous variables relative to demographic and socio-economic status^(^ ^37^ ^)^. For the TDS2 study, a complex food sampling plan covering 90 % of the French diet was designed, taking into account the seasonal nature of products and the regional variations, leading to an accurate assessment of the population exposure at the national level^(^ ^50^ ^)^. In addition, the use of factorial analysis raises some concerns about the degree of subjectivity involved in the analytical process (e.g. the determination of the number of CS, the level of aggregation of foods, the determination of the number of patterns identified). However, as highlighted in Newby & Tucker^(^ ^80^ ^)^, the consistency and reproducibility with regard to other national and international studies help to confirm the validity of our findings.

In conclusion, from the INCA2 survey, we identified seven distinct dietary patterns in the French adult population, with specific demographic, socio-economic, nutritional and environmental characteristics. These findings provide new information on the diversity of food consumption in France and give an overview of the nutritional quality of the different food consumption behaviours. From a public health perspective, our results provide interesting insights for developing behaviourally targeted policies. In addition, because of contradictory results for a given dietary pattern between high-quality nutritional intake and high contaminant exposure levels (and vice versa), this study also demonstrates the necessity to analyse the risks and the benefits of food consumption behaviours, particularly in a public health context. Finally, the food consumption data were collected several years ago (i.e. 2006–2007) and the third INCA survey is currently underway, potentially providing the opportunity to assess the trends in dietary patterns at the national level.

## Supplementary material

For supplementary material accompanying this paper visit http://dx.doi.org/10.1017/S0007114516001549. click here to view supplementary material
