# Supplementary material for: Dietary patterns in the French adult population: a study from the second French national cross-sectional dietary survey (INCA2) (2006–2007)
Source: Br J Nutr. 2016 May 18;116(2):300–15. doi: 10.1017/S0007114516001549 (PMC4910537; doi:10.1017/S0007114516001549)
Supplement: Supplementary file 1 [file S0007114516001549sup001.docx]

**SUPPLEMENTARY MATERIAL**

[Table S1. Socio-demographic characteristic of adults from INCA2 study and comparison with French national data INSEE 2005 (18-79yo)† 2](#_Toc443734266)

[Table S2. Demographic, socio-economic variables 3](#_Toc443734267)

ANNEXES

1. Socio-demographic characteristic of adults from INCA2 study and comparison with French national data INSEE 2005 (18-79yo)†

|  | **Repartition (%)** | **Repartition (%)** |  |
| --- | --- | --- | --- |
|  | **INCA2, adults**†  (N=2624) | **Labour force survey 2005-INSEE**  (N=44 368 541) | **p**‡ |
| **Gender** |  |  | <0.001 |
| Men | 41.4 | 48.6 |  |
| Women | 58.6 | 51.4 |  |
| **Age** |  |  | <0.001 |
| 18-24 yo. | 9.8 | 12.4 |  |
| 25-34 yo. | 16.5 | 18.0 |  |
| 35-49 yo. | 31.9 | 29.0 |  |
| 50-64 yo. | 28.6 | 24.6 |  |
| 65-79 yo. | 13.3 | 16.0 |  |
| **Household size** |  |  | <0.001 |
| 1 person | 21.2 | 14.3 |  |
| 2 persons | 35.7 | 33.8 |  |
| 3 persons | 16.6 | 20.4 |  |
| 4 persons | 16.4 | 20.1 |  |
| 5 and more persons | 10.2 | 11.5 |  |
| **Occupational status** |  |  | <0.001 |
| Farmer | 2.5 | 2.1 |  |
| Artisans, merchant and chief manager | 4.8 | 5.6 |  |
| Higher managerial and professional occupations | 12.3 | 12.2 |  |
| Intermediate occupations | 16.3 | 15.2 |  |
| Employee | 15.7 | 10.7 |  |
| Worker | 18.9 | 21.5 |  |
| Former working person | 23.8 | 29.6 |  |
| Other working person | 5.6 | 3.2 |  |

† Repartition of the socio-demographic characteristics before adjustment

‡ Chi-squared contingency table test

1. Demographic, socio-economic variables

| **Variable** | **Levels** |
| --- | --- |
| Age | 18-24 yo, 25-34 yo, 35-49 yo; 50-64 yo; 65-79 yo |
| Sex | Male, Female |
| Occupational status | Low (trade workers, operators, labourers, unemployed workers)  Medium (farmers, service and sales workers, technicians, etc.)  High (executives, managers, professionals)  Economically inactive (student, housewife, retired…) |
| Household income | <€1299/month, between €1300 and €1899/month, between €1900 and €2499/month, between €2500 and €4599€/month, ≥ €4600/month |
| Wealth Index | 1^st^ tertile, 2^nd^ tertile, 3^rd^ tertile |
| Number of people in the household | 1-2, 3-4, ≥ 5 |
| Composition of the household | Couple with children, Couple without children, Single-parent family, Single without children |
| Region of residence | Paris, north-west, north-east, south-west, south-east |
| Degree of urbanisation | Rural (2000 to 20 000 inh.), Small and medium town (20,000 to 100 000 inh.), Large town or city (> 100 000 inh.) |
| Educational level | Primary, Secondary, Post-secondary school |
| Food insecurity | No, Yes |
